# Choledochal Stenting for Treatment of Extrahepatic Biliary Obstruction in Dogs with Ruptured Gallbladder: 2 Cases

**DOI:** 10.3390/vetsci12070673

**Published:** 2025-07-17

**Authors:** Shin-Ho Lee, Jeong-Hyun Seo, Jae-Hyeon Cho

**Affiliations:** 1 Department of Companion Animal Health, Tongmyong University, Busan 48520, Republic of Korea; hovet519@naver.com; 2 Time Animal Medical Center, Daejeon 35233, Republic of Korea; 3 Institute of Animal Medicine, Gyeongsang National University, Jinju 52828, Republic of Korea

**Keywords:** dog, extrahepatic biliary obstruction, choledochal stenting, pancreatitis, treatment

## Abstract

Two geriatric (>9 years old) dogs presented with vomiting, depression, and anorexia and were diagnosed with extrahepatic biliary obstruction (EHBO) secondary to ruptured gallbladder mucoceles. Diagnosis was based on serum biochemical analysis, abdominal radiography, and ultrasonography, which revealed gallbladder rupture, peritonitis, and common bile duct dilation. Both dogs underwent emergency surgical intervention involving cholecystectomy and choledochal stent placement in the common bile duct without cholecystojejunostomy or cholecystoduodenostomy. Postoperatively, the clinical symptoms and serum chemistry values improved, and both dogs survived without recurrence for over one year. These cases demonstrate that choledochal stenting can be an effective adjunct to cholecystectomy for managing EHBO in dogs with ruptured gallbladder mucoceles, potentially preventing reocclusion and promoting recovery, especially when histopathological evaluation is not feasible in clinical settings. However, persistent elevation of liver enzymes may occur postoperatively, necessitating prolonged monitoring and medical management in some cases.

## Simple Summary

This report describes two older dogs with severe bile duct obstruction and ruptured gallbladders, leading to life-threatening illness. Both dogs were treated with emergency surgery to remove the damaged gallbladder and place a stent in the common bile duct to keep it open. After surgery, both dogs recovered well and lived for more than a year without the symptoms returning. The use of a stent in addition to gallbladder removal helped prevent the bile duct from becoming blocked again and supported the recovery. This approach may be a valuable option for dogs with similar conditions, although some may require ongoing monitoring of liver health.

## 1. Introduction

Gallbladder mucocele is a condition in which abnormally viscous mucin is secreted by the epithelial cells of the gallbladder and accumulates in the gallbladder, causing the bile to turn into a jelly-like substance that can cause the gallbladder to distend and lead to serious complications such as biliary obstruction, gallbladder wall ischemia, necrosis, rupture, and biliary peritonitis [1].

External biliary obstruction in dogs is a rare disease caused by gastrointestinal diseases including bile sludge, gallstones, pancreatitis, tumors, and gallbladder mucosa [2]. The clinical features and prognostic factors of extrahepatic biliary obstruction (EHBO) secondary to pancreatitis have been analyzed in a cohort of 46 dogs. This study evaluated survival rates and identified risk factors associated with poor outcomes in affected dogs [3].

Furthermore, there have been reports indicating that the presence of gallstones in the gallbladder can cause a blockage in the bile duct, leading to EHBO and rupture of the gallbladder [4]. Successful surgical methods such as cholecystojejunostomy and cholecystoduodenostomy have been used for the treatment of EHBO. However, these procedures can result in normal changes in EHBO and may be associated with several complications [5]. Common bile duct stents are used in veterinary medicine to manage obstructive biliary disease by decompressing the biliary tract before surgery [6]. This study aimed to describe the clinical pathological characteristics, diagnosis using abdominal ultrasonography, and surgical treatment and results through stent-equipped cases in the common bile duct to treat two dogs with gallbladder mucosa and EHBO rupture.

## 2. Materials and Methods

### 2.1. Case I

An 8-year-old, 5.8 kg, neutered male Pomeranian was diagnosed with bladder stones, gallbladder inflammation, and gallbladder mucus two months before visiting and re-presented to the hospital due to severe vomiting, loss of energy, and loss of appetite. No specific findings were observed in the complete blood count (CBC, Procyte Dx, Westbrook, ME, USA). The preoperative serum chemical test (Catalyst One chemistry analyzer, Westbrook, ME, USA)results are shown in Table 1.

**Figure 1:**
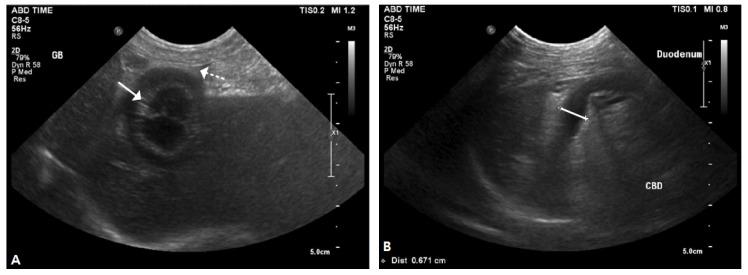
Gallbladder mucocele and distension of the common bile duct on abdominal ultrasonography. (**A**) Exudate and highly echogenic lipids were found around the gallbladder (dashed arrow with dotted line) and the gallbladder was filled with tacky mucus (solid arrow). (**B**) The common bile duct was markedly dilated (the line length was 0.671 mm).

**Figure 2:**
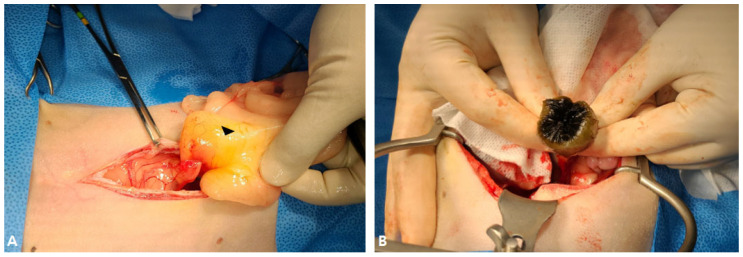
Fatty tissue connected to the falciform ligament and tacky mucus in the gallbladder. (**A**) Yellowish fluid was identified in the adipose tissue connected to the falciform ligament. (**B**) Gelatinous mucus was observed in the gallbladder.

In addition, a positive result was confirmed using the Canine Pancreatic Lipase (cPL) kit test. Abdominal ultrasonography revealed a thickened gallbladder wall. The lumen contained many immobile high-echo substances, most of which were attached to the gallbladder wall, and it appeared to be a kiwi-shaped mucous formation. A wide range of bile peritonitis can identified by echo elevation in the hepatic parenchyma, fat around the gallbladder, and the identification of exudates around the gallbladder (Figure 1A). The lumen of the common bile duct was expanded by 0.671 mm. The average diameter of the canine common bile duct is 3 mm (Figure 2B) (Long, 2004). Adhesion to the surrounding fat and liver lobe, as well as gas in the common bile duct, was identified, and common bile duct rupture due to EHBO and bile outflow was suspected.

Based on clinical signs, the clinical pathological findings, and imaging diagnosis results, the primary differential diagnoses were cholecyst rupture, EHBO, and peritonitis caused by gallbladder mucocele. The patient was hospitalized, and maropitant (1 mg/kg, q24 h, Cerenia^®^, Zoetis, Florham Park, NJ, USA), cephazolin (25 mg/kg, q12 h, cefazolin Inj. Chong Kun Dang Pharm, Seoul, Republic of Korea), and tramadol (2 mg/kg, q12 h, Tridol Inj. Wuhan Corp, Republic of Korea) were intravenously administered for supportive therapy. Subsequently, emergency surgery was performed. Midazolam (0.85 mg/kg, Bukwang Midazolam Inj. Bukwang Pharmcorp., Seoul, Republic of Korea) and propofol (10 mg/kg, Provive, Myungmoon Pharm, Seoul, Republic of Korea) were administered for the induction procedure. After intubation using an endotracheal tube (Rushelit, size ID 3.5 mm, OD 5.3 mm, Teleflex, Kulim, Malaysia), general anesthesia using isoflurane (Ifran, Hana Pharm, Seoul, Republic of Korea) was induced by forced breathing circulation using a respiratory anesthetic machine (Drager primus, Dragerwerk AG & Co. KGaA, Lübeck, Germany) with a volume of 50–60 cc. The patient was allowed to lie in the dorsal recumbency position, the entire abdomen was clipped from the lower chest, and general disinfection was performed for surgery. The median line was incised from the processus xiphoideus, and traces of exposure to bile acid were found in the adipose tissue connected to the falciform ligament, which was removed to secure visibility for surgery (Figure 2A). Furthermore, the surgical field of view was secured using a Balfour abdominal retractor. A previously ruptured gallbladder was identified, and the body was filled with kiwi-shaped mucus (Figure 2B).

**Figure 3:**
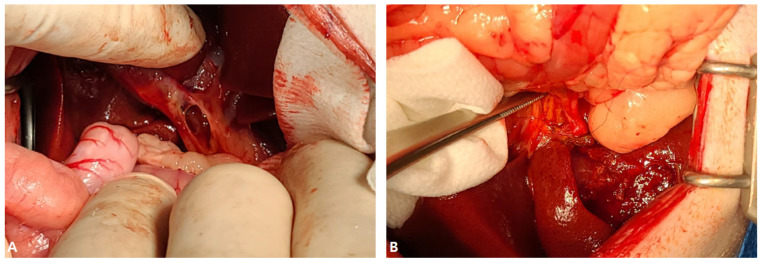
Suture of a ruptured common bile duct. (**A**) Vertically ruptured common bile duct was identified. (**B**) The ruptured common bile duct was sutured with a simple continuous suture using absorbable sutures.

To perform the cholecystectomy, the gallbladder was gently pulled using mezenbaum and electrocautery, and double ligation was performed in the proximal part of the bile duct separated from the right lobe and quadrate lobes of the liver by an absorbable suture (3-0 polydioxanone, PDS^®^ II, Ethicon, Johnson and Johnson, Somerville, MA, USA). After the gallbladder was removed, the ruptured common bile duct was explored (Figure 3A).

The ruptured common bile duct was sutured by absorbable sutures (6-0 polydioxanone, PDS^®^ II, Ethicon, Johnson and Johnson, Somerville, MA, USA) with a simple continuous suture (Figure 3B). Then, at the 5 cm point of the distal part of the pyloric part, the duodenum was cut on the opposite side of the mesentery, and a 5 French feeding tube (F.D.T(D) 5Fr-500, HMS Inc., Uiwang, Republic of Korea) was inserted into the papilla where the bile sludge was visible. It was washed three times using a feeding tube with sterilized saline to remove sludge from the remaining bile, and the tube was cut into 6 cm and installed through the mucous membrane of the duodenum with a simple interrupted suture (4-0 polydioxanone, PDS^®^ II, Ethicon, Johnson and Johnson, Somerville, MA, USA). The duodenum was closed with a simple continuous suture (4-0 polydioxanone, PDS^®^ II, Ethicon, Johnson and Johnson, Somerville, MA, USA). The abdominal cavity was washed sufficiently with sterile saline. The dog was then routinely sutured in the order of muscle, subcutaneous, and skin, and a drainage tube (BAROBAC^®^, SEWOON MEDICAL CO, Seoul, Republic of Korea) was installed to remove blood substances and bile acid remaining in the abdominal cavity due to bile peritonitis.

For postoperative care, the patient was administered cefazolin (22 mg/kg, IV, q12 h) as an antibiotic, and the pain was treated with tramadol (2 mg/kg), and a combination of tramadol (1.3 mg/kg/h), lidocaine (3 mg/kg/h, 2%, Jeil Pharmaceutical Co., Seoul, Republic of Korea), and ketamine (0.6 mg/kg/h, Ketamine HCl Injection Huons, HUONS Co., Seongnam, Republic of Korea) was administered by constant rate infusion. The postoperative serum chemical test results are shown in Table 2.

On the third day after surgery, only 1 to 3cc of blood substances were drained and removed from the drainage tube, and the dog had good vital signs but no voluntary eating. Forced feeding was performed. Tramadol (2 mg/kg, q12 h, Tridol Cap, Yuhan Corp, Seoul, Republic of Korea), amoxicillin-clavulanic acid (22 mg/kg, q12 h, bid, Amocla Tab, KUHNIL Corp, Seoul, Republic of Korea), metronidazole (15 mg/kg, q12 h, bid, Flasinyl Tab, HK inno.N Corp, Cheongju, Republic of Korea), gabapentin (10 mg/kg, q12 h, Gabalep Cap., Chong Kun Dang Pharmaceutical Corp., Seoul, Republic of Korea), streptokinase (0.05 T/kg, q12 h, Verase Tab., Nelson, Seoul, Republic of Korea), misoprostol (5 ug/kg, q12 h, Misoprostol Tab., Nelson, Seoul, Republic of Korea), ursodeoxycholic acid (10 mg/kg, q12 h, Ursa Tab, Daewoong Pharmaceutical Co., Seoul, Republic of Korea) and Silymarin (10 mg/kg, q12 h, Silymarin Tab, Sinil Pharm Co., Chungju-si, Republic of Korea) were prescribed. On the fourth day after surgery, the dog started eating voluntarily, and the vital signs improved; therefore, the dog was discharged from the hospital and presented to the hospital for liver function tests and improvement. ALKP and GGT levels continued to be higher than the reference range for five months after surgery; however, vital signs, defecation, urination, and appetite were good, and both ALKP and GGT were confirmed to be in the normal range at 6 months after surgery. The owner also did not mention any symptoms related to EHBO, and treatment was terminated. One month after surgery, the stent was excreted in the dog’s feces.

### 2.2. Case II

A Pomeranian, a 3.3 kg, 10-year-old neutered female, was admitted to the hospital due to vomiting and loss of appetite. Physical examinations showed discomfort in vocalization and avoidance during abdominal palpation. The preoperative CBC and serum chemical test results are shown in Table 3.

**Figure 4:**
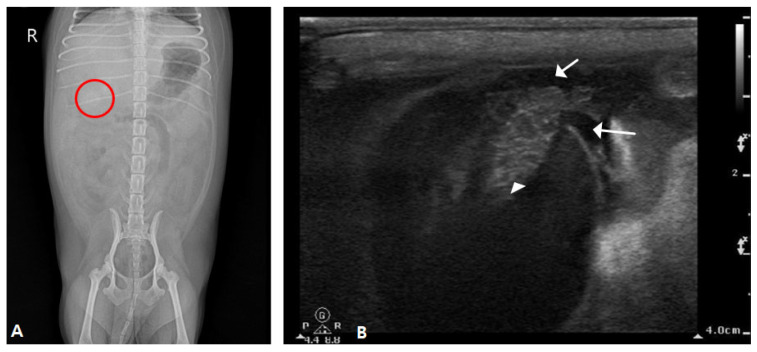
Radiography and sonography of the gallbladder. (**A**) An opaque material with rounded margins was identified in the right upper abdomen (round circle). (**B**) Hyperechoic round-shaped immobile sludge inside the gallbladder penetrated the abdominal gallbladder wall (arrowhead) and spanned the wall, and free fluid was identified around the gallbladder (arrow).

Abdominal radiography revealed mineralized radiopaque substances in the right upper abdomen (Figure 4A). Abdominal ultrasonography revealed dilatation of the gallbladder with an irregular, immobile substance inside the gallbladder that penetrated the ruptured gallbladder wall. In addition, a large amount of echogenic ascites were confirmed around the gallbladder, fat thickening was confirmed, and peritonitis was suspected (Figure 4B).

In conclusion, the patient was diagnosed with peritonitis, gallbladder mucosa, and gallbladder rupture, and emergency surgery was performed. The entire anesthesia process, drug type and dose, and disinfection procedure for surgery were performed in the same manner as in case 1.

**Figure 5:**
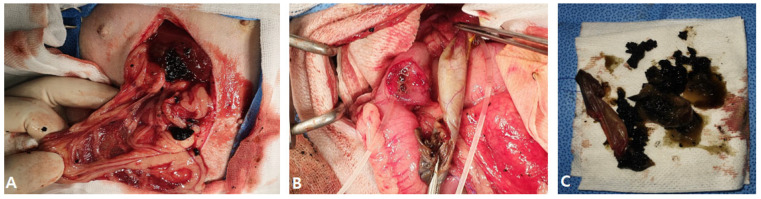
Severe peritonitis caused by a ruptured gallbladder and choledochal stent in the duodenum. (**A**) Along with peritonitis, sludge and fragments of the gallbladder had leaked, and the liver was enlarged. (**B**) The choledochal tube was stented for decompression of the extrahepatic portion of the biliary tract. (**C**) A large amount of tacky mucus was identified in the ruptured gallbladder.

An incision was made from the processus xiphoideus to the midline of the abdomen. Upon opening, large and small green gallbladder sludge and debris with bleeding were observed, and the liver was enlarged (Figure 5A). The ruptured gallbladder was found and retracted, and a duodenal incision was made on the opposite side of the mesenteric membrane to visualize the duodenal papilla. A stent was installed through the duodenal papilla using a 6 French feeding tube (Figure 5B).

3-0 PDS^®^II was installed in the lumen by suturing through the submucosa with a simple interrupted suture. Through the installed stent, the remaining sludge in the common bile and hepatic ducts was removed three times by flushing with sterile saline, and patency was secured. The incised duodenum was simply interrupted using 3-0 PDS^®^II. Double ligation using an absorbent suture of 3-0 PDS^®^II was applied and the ruptured bile duct was removed (Figure 5C). In addition, extensive washing was performed using sterile saline in the abdominal cavity, and the surgery was completed with routine suturing.

Immediately after surgery, tramadol, lidocaine, and ketamine were administered in the same manner as in case 1 to control pain, and on the third day, pain management was stopped due to no pain response, good appetite, and stable vital signs. Management of peritonitis was conducted with cefixime (10 mg/kg, q12 h, Nelson, Seoul, Republic of Korea), metronidazole (15 mg/kg, q12 h, Flasinyl Tab, HK inno.N Corp, Cheongju, Republic of Korea), and streptokinase (0.05 T/kg, q12 h, Verase Tab., Nelson, Seoul, Republic of Korea) 0.05 tablets/kg PO BID before and after discharge. The presence of the stent could not be confirmed after the patient was discharged.

Defecation, urination, and appetite were normal. Sutures were removed two weeks after surgery, and all serum chemical test results were confirmed to be within the normal range. Table 4 presents the CBC and serum chemistry results from preoperative days 3 and 6.

## 3. Discussion

Bile sludge moves according to the position of the dog without acoustic shading on abdominal ultrasound and is not related to biliary tract disease [7]. However, gallbladder mucus does not move as the patient’s position changes, and the bile is concentrated and extends linearly from the gallbladder wall to the lumen, or the marginal area is anechoic, and the center of the mucus is highly echoic [1]. In this study, a star shape or cut kiwi shape in the gallbladder lumen was identified in case 1, but not in case 2. Both cases were immobile, highly echogenic substances were imaged, and there was no change depending on the patient’s position; therefore, it was distinguished from normal sludge.

A previous study also emphasized that, in gall bladder mucus, gravity-independent mucus adherent to the gallbladder wall and protruding into the lumen is observed on ultrasonography, and the immobility of this material—unlike typical bile sludge—is key to diagnosis [1]. This mucus often exhibits a linear or stellate pattern, with the center appearing hyperechoic. In addition, only twelve (63%) of nineteen dogs with EHBO had a distended bile duct. In this case, discontinuity of the gallbladder wall was confirmed, and peritonitis and ascites, which are inflammatory findings of high echogenic fat, were observed around the gallbladder, and gallbladder rupture was suspected. In addition, because of the distended common biliary tract, the possibility of EHBO was considered, and it was approached as an emergency surgical intervention. Pancreatic nodules were observed in 80.2% of the 101 dogs, and these nodules most commonly appeared in association with the histopathological features of chronic pancreatitis, such as fibrosis, atrophy, and lymphocytic inflammation [8]. Pancreatitis is a common cause of EHBO in dogs [9]. Of the 19 animals that underwent surgical treatment, 18 (95%) survived, including 1 animal who also underwent a stent insertion [2]. Biliary decompression can be achieved through surgical interventions, such as percutaneous ultrasound-guided cholecystocentesis, retrograde biliary stent installation using endoscopes, cholecystoduodenostomy, and choledochal stent installation [3,10].

In two cases, intra-pancreatic nodules were confirmed on abdominal ultrasound, chronic pancreatitis was suspected, gallbladder rupture due to EHBO was confirmed during surgery and on abdominal ultrasound, and serious luminal mucus was observed. The presence of pancreatic nodules can be interpreted as an indicator of chronic pancreatitis or chronic pancreatic injury, representing the result of prolonged, repetitive inflammation and tissue regeneration, rather than acute pancreatitis. Although secondary EHBO occurred, the serum chemistry levels were normalized, and clinical symptoms clearly improved within 6 days of surgery with stent placement. However, in this case, since there are no histopathological results, it is not possible to clearly determine whether it is acute or chronic pancreatitis. Histopathological results are unrealistic in the clinical environment, and pancreatitis often has its own limitations because it has a partial distribution, and it is difficult to determine the clinical relevance of inflammatory lesions. Unlike case 2, in case 1, clinical symptoms occurred two months ago, and without surgical intervention, the dog was presented to the hospital due to vomiting, jaundice, and loss of appetite.

In a retrospective study of 89 dogs diagnosed with gallbladder mucoceles, gallbladder mucoceles type, serum alkaline phosphatase activity, and serum creatinine and phosphorus concentrations were associated with decreased survival across groups [11]. In case 1, the total bilirubin level was higher than the reference range in case 2. Clinical symptoms improved in both cases after surgery; however, in case 1, ALKP and GGT levels were elevated for five months after cholecystectomy and stent installation, and medical management was required.

Five cases of unjaundiced gallbladder rupture were reported in serum chemistry tests, with an average TB of 0.2 mg/dL (normal range, 0–0.9 mg/dL), and as a result of bile acid analysis through abdominocentesis in one dog, the same day, the concentration of the bile significantly increased. The concentration of bilirubin in the ascites of the patient was 0.4 mg/dL [12]. According to a previous study, out of twenty-two cases of gallbladder mucosa, nine individuals had jaundice during physical examination, and seventeen cases confirmed an increase in TB [13]. If a mucocele is present in the gallbladder and serum chemical analysis shows low levels of TB, it indicates that only a small amount of mucus is stored in the gallbladder. In other situations, even if a rupture occurs, the amount of bile leaking into the surrounding area is minimal. Alternatively, it could be an unjaundiced gallbladder rupture that did not have a high TB level because the rupture had occurred recently. Surgical decompression is known to rapidly restore bile flow, reduce the risk of bile leakage and sepsis, and promote hepatic regeneration and recovery, while postoperative monitoring of TB levels is useful for the early detection of biliary re-obstruction, stent occlusion, or hepatic dysfunction [14]. In both cases 1 and 2, TB decreased rapidly after surgery, suggesting that recovery was also rapid.

The primary purpose of stent installation is to prevent anatomical damage caused by inflammation, ulcers, and edema in the bile duct due to acute pancreatitis [15]. This allows for proper bile outflow and gives time for the damaged structure to recover. In both case 1 and case 2, pancreatitis was treated by installing a stent to prevent re-occlusion. Following the surgery, the pancreas slowly recovered and the clinical symptoms showed improvement.

Gallbladder rupture is common, and rapid surgical intervention following gallbladder rupture can produce an excellent prognosis [16]. The cause of gallbladder mucocele, which results in persistent elevation of liver enzymes in some dogs even after surgery, is still not fully understood. In both cases 1 and 2, surgical intervention was performed urgently upon the diagnosis of cholecyst rupture in abdominal ultrasound. Both cases were discharged with good appetite from the hospital on the fourth day after surgery. Regarding case 1, the vomiting and diarrhea have ceased and the owner reports that the vital signs are excellent. However, the liver levels have been consistently increasing, necessitating monitoring for a period of five months. The cause of gallbladder mucocele cysts remains unknown [14]. However, surgical procedures such as cholecystectomy or cholecystoduodenostomy are effective in treating this condition. Removal of the gallbladder also eliminates the risk of biliary wall necrosis. Stents were installed in the common bile duct after three days in EHBO progressed to chronic cholangitis after surgery in dogs with gallbladder mucocele removed [16]. Considering that EHBO occurred due to chronic cholangitis after cholecystectomy in gall bladder mucocele, it was considered that cholecystectomry and washing the common bile duct may not be enough to prevent the occurrence of EHBO. In case 2, the patient was discharged from the hospital and stent discharge could not be confirmed. Regular follow-up imaging such as abdominal X-ray or CT scan is recommended to monitor the location and status of the stent and to promptly identify potential complications. Additionally, close clinical follow-up should be maintained to check for symptoms suggestive of stent migration or obstruction.

As seen in this case, there may be chronic changes in the liver, hepatic duct, and common bile duct in individuals with gallbladder rupture, so installing a stent can reduce the occurrence of EHBO.

## 4. Conclusions

Two dogs were diagnosed with cholecyst rupture, and emergency surgical procedures were performed which involved cholecystectomy and choledochal stent installation. Diagnosing gallbladder rupture can be challenging due to various factors such as echogenic fat content around the gall bladder, ascites, and discontinuity in the gallbladder wall. However, if there is also echogenic sediment in the gallbladder, it may indicate gallbladder rupture due to gallbladder wall necrosis. As a clinical limitation, histopathological examinations of the biliary tract and the duodenal papilla are difficult, and re-occlusion may occur after cholecystectomy, so choledochal stent installation can be said to be a way to prevent re-occlusion of the biliary tract and increase the possibility of healing.

## Figures and Tables

**Table 1:** Preoperative serum chemical analysis.

Test Item	Measured Value	Reference Range
Album	3 g/dL	2.3–4.0 g/dL
Alanine Transferase (ALT)	401 U/L	10–125 U/L
Alkaline Phosphatase (ALKP)	1.888 U/L	23–212 U/L
Gamma-glutamyl Transferase (GGT)	11 U/L	0–11 U/L
Total bilirubin (TB)	3.1 mg/dL	0–0.9 mg/dL
C-reactive Protein (CRP)	50 mg/L	0–1 mg/dL

**Table 2:** Postoperative serum chemical analysis.

Test Item	Measured Value	Reference Range
Album	2.3 g/dL	2.3–4.0 g/dL
Alanine Transferase (ALT)	155 U/L	10–125 U/L
Alkaline Phosphatase (ALKP)	1.944 U/L	23–212 U/L
Gamma-glutamyl Transferase (GGT)	23 U/L	0–11 U/L
Total bilirubin (TB)	0.4 mg/dL	0–0.9 mg/dL
C-reactive Protein (CRP)	13.6 mg/L	0–1 mg/dL

**Table 3:** Preoperative CBC and serum chemical analysis.

Test Item	Measured Value	Reference Range
White Blood Cell Count (WBC)	24.34 × 10^9^/L	5.05–16.76 × 10^9^/L
Albumin	2.1 g/dL	2.3–4.0 g/dL
Alanine Transferase (ALT)	437 U/L	10–125 U/L
Alkaline Phosphatase (ALKP)	1.659 U/L	23–212 U/L
Gamma-glutamyl Transferase (GGT)	27 U/L	0–11 U/L
Total bilirubin	2.4 mg/dL	0–0.9 mg/dL
C-reactive Protein (CRP)	13.6 mg/L	0–1 mg/dL
Canine Pancreatic Lipase (cPL)	1.4353 ng/mL	200–400 ng/mL

**Table 4:** Comparison between postoperative day 3 and day 6.

Test Item	Postoperative Day	Measured Value	Reference Range
Albumin	Day 3	2.3 g/dL	
	Day 6	3.6 g/dL	
Alanine Transferase (ALT)	Day 3	193 U/L	10–125 U/L
	Day 6	122 U/L	
Alkaline Phosphatase (ALKP)	Day 3	1.633 U/L	23–212 U/L
	Day 6	624 U/L	
Gamma-glutamyl Transferase (GGT)	Day 3	12 U/L	0–11 U/L
	Day 6	7 U/L	
Total bilirubin (TB)	Day 3	0.8 mg/dL	0–0.9 mg/dL
	Day 6	0.5 mg/dL	
C-reactive Protein (CRP)	Day 3	5.9 mg/L	0–1 mg/dL
	Day 6	3.7 mg/L	
Canine Pancreatic Lipase (cPL)	No record	No record	200–400 ng/mL
	Day 6	86.1 ng/mL	

## Data Availability

No new data were created or analyzed in this study. Data sharing is not applicable to this article.
